# In silico engineering and simulation of RNA interferences nanoplatforms for osteoporosis treating and bone healing promoting

**DOI:** 10.1038/s41598-023-45183-3

**Published:** 2023-10-24

**Authors:** Aylar Imanpour, Hanieh Kolahi Azar, Dorna Makarem, Zeinab Nematollahi, Reza Nahavandi, Mohammadreza Rostami, Nima Beheshtizadeh

**Affiliations:** 1 Regenerative Medicine Group (REMED), Universal Scientific Education and Research Network (USERN), Tehran, Iran; 2 Department of Pathology, Tabriz University of Medical Sciences, Tabriz, Iran; 3 Escuela Tecnica Superior de Ingenieros de Telecomunicacion, Politecnica de Madrid, Madrid, Spain; 4 UCL Department of Nanotechnology, Division of Surgery and Interventional Science, University College London, London, UK; 5 Department of Biochemical and Pharmaceutical Engineering, School of Chemical Engineering, College of Engineering, University of Tehran, Tehran, 11155-4563 Iran; 6 Food Science and Nutrition Group (FSAN), Universal Scientific Education and Research Network (USERN), Tehran, Iran; 7 Division of Food Safety and Hygiene, Department of Environmental Health Engineering, School of Public Health, Tehran University of Medical Sciences, Tehran, Iran; 8 Department of Tissue Engineering, School of Advanced Technologies in Medicine, Tehran University of Medical Sciences, Tehran, Iran

**Keywords:** Gene ontology, Biomedical engineering, Bone, Tissue engineering and regenerative medicine

## Abstract

Osteoporosis is a bone condition characterized by reduced bone mineral density (BMD), poor bone microarchitecture/mineralization, and/or diminished bone strength. This asymptomatic disorder typically goes untreated until it presents as a low-trauma fracture of the hip, spine, proximal humerus, pelvis, and/or wrist, requiring surgery. Utilizing RNA interference (RNAi) may be accomplished in a number of ways, one of which is by the use of very tiny RNA molecules called microRNAs (miRNAs) and small interfering RNAs (siRNAs). Several kinds of antagomirs and siRNAs are now being developed to prevent the detrimental effects of miRNAs. The goal of this study is to find new antagonists for miRNAs and siRNAs that target multiple genes in order to reduce osteoporosis and promote bone repair. Also, choosing the optimum nanocarriers to deliver these RNAis appropriately to the body could lighten up the research road. In this context, we employed gene ontology analysis to search across multiple datasets. Following data analysis, a systems biology approach was used to process it. A molecular dynamics (MD) simulation was used to explore the possibility of incorporating the suggested siRNAs and miRNA antagonists into polymeric bioresponsive nanocarriers for delivery purposes. Among the three nanocarriers tested [polyethylene glycol (PEG), polyethylenimine (PEI), and PEG-PEI copolymer], MD simulations show that the integration of PEG-PEI with has-mIR-146a-5p is the most stable (total energy = -372.84 kJ/mol, Gyration radius = 2.1084 nm), whereas PEI is an appropriate delivery carrier for has-mIR-7155. The findings of the systems biology and MD simulations indicate that the proposed RNAis might be given through bioresponsive nanocarriers to accelerate bone repair and osteoporosis treatment.

## Introduction

Osteoporosis, a metabolic bone disease characterized by poor bone density and degeneration of bone architecture that increases the risk of fractures, affects about 10 million men and women in the United States^1^. Fractures caused by osteoporosis may increase pain, disability, skilled nursing assignments, overall health care expenses, and death^2^. The primary method for diagnosing osteoporosis is to measure bone mineral density (BMD) employing non-invasive dual-energy x-ray absorptiometry. Therapy for osteoporosis includes bisphosphonates, inhibitors of the receptor activator of nuclear factor kappa-B ligand, estrogen agonists/antagonists, parathyroid hormone analogues, and calcitonin^3,4^. A cathepsin K inhibitor and a monoclonal antibody against sclerostin are two emerging drugs that use unique techniques to treat osteoporosis^5,6^.

As per a prior investigation of the cost-effectiveness literature concerning the effectiveness of oral bisphosphonates, it has been determined that alendronate and risedronate exhibit the highest degree of cost-effectiveness in females with low bone mineral density who have not undergone prior fractures^7^. There exist variations in the guidelines pertaining to the administration of denosumab for therapeutic purposes. In economic evaluations conducted on therapy for postmenopausal women, Denosumab outperformed risedronate and ibandronate. However, despite being equally effective as generic alendronate, it incurred higher costs^8^. According to a study, Denosumab was found to be a more cost-effective option than bisphosphonates and teriparatide for the treatment of osteoporosis in older males^9^. In addition to its status as the primary cause of fractures among the elderly, osteoporosis exhibits a significant correlation with prolonged periods of bedrest, thereby increasing the likelihood of severe outcomes^10^. Major therapeutic discoveries in osteoporosis therapy have been achieved in recent years as researchers obtain better knowledge of bone shape and the underlying processes that cause osteoporosis.

However, the long-term use of these therapies is limited due to side effects such as gastrointestinal intolerance, osteonecrosis, over-suppression of bone turnover, thromboembolic disease, and increased cancer risk^11^. Therefore, there is an urgent need to develop novel anti-osteoporotic drugs that are safer, more effective, and have a wider therapeutic window, while minimizing side effects.

Bone regeneration therapies hold potential for treating complex bone fractures by restoring the function of damaged cells or tissues^12^.﻿ Cytokines and growth factors, like bone morphogenetic proteins (BMPs), are widely employed to enhance the regenerative properties of materials. However, the clinical use of recombinant osteogenic proteins is hindered by their instability, high cost, and short lifespan^11^. Consequently, alternative approaches are necessary to enhance the effectiveness of bone regeneration materials.

In this regard, engineering ribonucleic acid interference (RNAi), which is a fundamental biological process that regulates gene expression at the post-transcriptional level, could be considered^13,14﻿^. It involves the specific silencing of target mRNA molecules through a sequence-specific mechanism. The RNAi-utilizing method is based on two kinds of small RNA molecules: microRNA (miRNA) and small interfering RNA (siRNA)^15^. miRNAs are single-stranded (18–23 nucleotide) small non-coding endogenous products that bind to targeted miRNAs and degrade or block gene translation^16^. As a result, miRNAs may have a considerable influence on a variety of pathological and physiological processes. Because of their therapeutic potential, anti-miRNAs, or antagomirs, may be used to prevent the negative effects of miRNA^17^.

Along with miRNAs, siRNAs play a crucial role in the RNAi process that results in gene knockdown. The mechanism of gene silencing by siRNAs is more selective than that of miRNAs, which often affect numerous target genes^18^. Synthetic siRNAs have emerged as potential therapeutic agents for a variety of diseases, including cancer, metabolic disorders, inflammatory disorders, and infectious diseases^19^. Due to the variations in techniques, the use of siRNAs and miRNAs in pharmaceutical applications may be regarded as a combination or parallel treatment. siRNA molecules are designed to degrade specific mRNA sequences, while antagomirs are modified RNA molecules that modulate the activity of miRNAs within the cell.

Several therapeutic techniques for siRNAs have been developed and gained traction in recent years^20^. In the context of osteoporosis and bone repair, siRNA targeting Runt-related transcription factor 2 (Runx2)/Core-binding factor alpha-1 (Cbfa1) has shown promise in inhibiting heterotopic ossification induced by bone morphogenetic protein 4 (BMP4), demineralized bone matrix, and trauma in animal models^21^. In addition, inhibition of SOST expression has been found to enhance osteoblast activity and promote bone formation^22^. These findings highlight the potential of siRNA-based strategies for preventing and treating abnormal bone formation and improving bone health.

The development of effective and safe delivery vehicles for therapeutic RNAi modulators is crucial for advancing RNAi technologies in the treatment of bone diseases^23^﻿. Polymer-based delivery systems, such as those utilizing polyethylene glycol (PEG), polyethyleneimine (PEI), and PEG-PEI, play a significant role in this field^24^. These systems offer distinct advantages and show promise in enhancing the efficacy and safety of RNAi modulator delivery. However, it is important to recognize that the behavior of specific RNAi modulators can vary when combined with different polymer-based delivery systems. The choice of polymer-based system is influenced by the specific characteristics and requirements of the RNAi modulators, including their size, charge, and stability.

Biological activity, on the other hand, is based on molecular interactions, which are the result of macromolecular structures^25^. Molecular dynamics (MD) simulations have evolved into an advanced method for identifying the relationships between macromolecular structure and function^26^. In addition, physiologically appropriate durations may be compared to simulation process timeframes. Knowledge of dynamic macromolecule characteristics is essential for structural bioinformatics to shift the paradigm from single-structure research to conformational ensemble assessment^27^. MD simulation, which can mimic biological macromolecules like proteins and genes as well as their interactions with diverse materials like polymeric nanocarriers, addresses prospective therapy methods for a variety of disorders.

The integration of systems biology and MD simulation is a powerful approach in understanding the behavior and stability of RNAi modulators in polymer-based delivery systems for the treatment of bone diseases. Systems biology enables the exploration of complex interactions and regulatory mechanisms associated with RNAi, while MD simulation provides insights into the structural dynamics and binding affinity of RNAi modulators and nanocarriers.

To date, several antagomirs and siRNAs have been proposed to mitigate the negative effects of miRNAs. To the best of our knowledge, no research has suggested antagomirs and siRNAs and discovered their transport using bioresponsive nanocarriers for bone healing and osteoporosis therapy. Almost little prior research has looked at the use of tailored and specialized small molecules in bone repair. The goal of this work is to offer novel antagonists for miRNAs and siRNAs, which decrease osteoporosis by targeting various genes and enhancing bone repair. Gene ontology techniques were utilized for this item by accessing several databases. The collected data was then subjected to a systems biology methodology. In addition, the integration of the proposed siRNAs and miRNAs with three different kinds of polymeric nanocarriers was studied using MD simulation.

## Materials and methods

### Functional enrichment analysis

To identify genes associated with bone regeneration, the regeneration gene database^28^ was utilized, resulting in the identification of 21 relevant genes. For the identification of genes associated with osteoporosis, the Disgenet database^29^ was employed with a score threshold of > 0.3, leading to the inclusion of 40 genes. The score threshold was set at > 0.3 to prioritize genes with a stronger association with osteoporosis, ensuring their relevance to the disease. This approach helped filter out genes with weaker or less significant associations, focusing on more robust candidates for further analysis. The gene ontology (GO) term and KEGG pathway enrichment^30,31^ analyses were conducted using the cluster profiler function package in the R language^32^. A significance threshold of p-value < 0.05 was applied to identify key pathways. The obtained results were visualized using the ggplot2 R package^33^.

### Construction and analysis of PPI and TFs network

The protein–protein interaction (PPI) network of the identified genes was constructed using the STRING database^34^. The resulting PPI network was visualized using Cytoscape v.3.9.0 software^35^. The network's topological characteristics were examined using the cytoHubba plug-in in Cytoscape. Additionally, the key genes within the PPI network were identified using the Molecular Complex Detection (MCODE) app^36^. To predict the transcription factors (TFs) associated with the candidate genes involved in osteoporosis and bone regeneration, the ChEA3 web server was employed^37^. ChEA3 utilizes the Fisher's Exact Test to determine the TFs that are closely associated with the input gene set^37^.

### Analysis by GeneMANIA

To gain further insights into the functions and potential interactions of the identified genes, we employed the GeneMANIA tool^38^. GeneMANIA is a powerful and user-friendly tool that allows for the analysis of gene lists and the generation of hypotheses regarding gene function. In our study, we inputted the names of the genes of interest and specified Homo sapiens as the study species. GeneMANIA leveraged comprehensive genomics and proteomics information to expand the gene clusters, identifying genes with similar functions or shared properties. The results of this analysis provided an interactive functional association network, visually illustrating the relationships among the genes and datasets. This network helped uncover potential functional interactions and provided valuable insights for further exploration of the genes' roles in osteoporosis and bone regeneration.

### Anti-miRNAs determining and siRNAs designing

To explore the regulatory mechanisms of the identified genes, we employed a combination of bioinformatics tools and databases. Firstly, the candidate genes were submitted to the Enrichr server^39^ for miRNA analysis. The Enrichr database provided valuable insights into the potential miRNAs that may target these genes. To validate the results obtained from Enrichr, we utilized the miRWalk database^40^, which incorporates multiple datasets, including TargetScan, miRDB, and miRTarBase, to generate predicted and validated miRNA-binding sites for known genes.

Next, we focused on the TFs that regulate the expression of specific miRNAs. The TransmiR database^41^ was employed to identify the TFs associated with miR-1277, miR-7155, miR-146a, miR-503, and miR-542. This database provided valuable information on the regulatory relationships between TFs and miRNAs. The resulting TF-miRNA regulatory network was visualized using the Cytoscape software, allowing us to better understand the interactions between miRNAs and their potential targets.

To inhibit the activity of the validated miRNAs, antisense RNAs, also known as anti-miRNAs or miRNA sponges were designed using the miRNAsong tool^42^. This web-based tool facilitated the generation of specific constructs that could effectively bind and sequester the targeted miRNAs. The sequences of HIF1A, IL1B, TNFSF11, and IL6 genes were retrieved from the NCBI database and utilized in the design process. Furthermore, to specifically target and silence the selected genes, siRNAs were designed using Eurofins Genomic's siRNA design tool^43^. The nucleotide sequences of each gene were aligned with nucleotide BLAST to ensure the safety and specificity of the designed siRNAs, minimizing the potential for off-target effects. By utilizing these computational tools and databases, we aimed to uncover potential miRNA-gene regulatory interactions and design effective anti-miRNAs and siRNAs for further analysis.

### Molecular dynamics simulation

To investigate the behavior of the polymeric nanocarriers and RNAi complexes, MD simulations were performed. The polymeric nanocarriers, consisting of 50 monomers, were designed using the CHARMM GUI^44^. The design process involved selecting the system type, specifying the monomer unit and number of monomers, determining the system size, performing equilibrium simulation, and generating input files for equilibration and production steps. The designed nanocarriers, including PEG, PEI, and PEG-PEI, served as crucial structures for subsequent molecular dynamics simulations. The RNAi structures were constructed using Maestro 12.8 from Schrodinger^45^. The force fields for the polymers and RNA were generated using the PolyParGen online tool^46^ and CHARMM GUI, respectively.

The all-atom MD simulations were conducted using GROMACS 2021^47^ with the OPLSA force field^48^. The nanocarriers in this study were intentionally designed to have a rod-like shape. This specific shape was selected due to its recognized advantages in nanocarrier design, particularly in terms of efficient encapsulation and delivery of cargo molecules. The elongated morphology of rod-shaped nanocarriers facilitates their ability to accommodate and transport cargo within their structures^49^. Each carrier-RNA complex was solvated in the SPCE water model with a concentration of 0.15 M NaCl. Periodic boundary conditions were applied, and the Particle Mesh Ewald (PME) method was used to calculate the electrostatic interactions. The temperature was maintained at 310 K, and the pressure was kept at 1 atm. Prior to the production runs, the systems underwent energy minimization using the steepest descent method for 5000 steps. Subsequently, the systems were equilibrated in the NVT (constant number of particles, volume, and temperature) and NPT (constant number of particles, pressure, and temperature) ensembles. The production runs were performed for 100 ns with a time step of 2 fs.

The root-mean-square deviation (RMSD), radius of gyration (Rg), and solvent-accessible surface area (SASA) were analyzed using the GROMACS 2021 software. The analysis of van der Waals (vdW), electrostatic, and total energy was performed using the mmpbsa command in GROMACS 2021. The visualization of the simulation results was carried out using VMD 1.9.4. By conducting MD simulations, we aimed to gain insights into the structural stability, conformational changes, and energetics of the polymeric nanocarriers and their interactions with RNAi. These simulations provided valuable information for evaluating the feasibility and efficacy of the proposed delivery system. All the steps and methods that are employed in this study are shown in Supplementary Fig. S1.

## Results

### GO and KEGG enrichment

GO and KEGG pathway enrichment analyses were performed to gain insights into the biological processes, molecular functions, cellular components, and pathways associated with bone regeneration (BRP) and osteoporosis (OSP). For BRP, 21 genes were identified from the regeneration gene database (Table 1), while 40 genes related to OSP with a score > 0.3 were obtained from the DisGeNET database (Table 2).

**Table 1:** The list of 40 genes, participated in the osteoporosis gathered from DisGeNET database.

No.	Name	Entrez ID	The gene whole name
1	WDR1	9948	WD repeat domain 1
2	PTH	5741	Parathyroid hormone
3	LRP5	4041	LDL receptor related protein 5
4	TNFRSF11B	4982	TNF receptor superfamily member 11b
5	PARK7	11315	Parkinsonism associated deglycase
6	ENO1	2023	Enolase 1
7	CYP19A1	1588	Cytochrome P450 family 19 subfamily A Member 1
8	ACTG1	71	Actin gamma 1
9	PRDX3	10935	Peroxiredoxin 3
10	ATIC	471	5-aminoimidazole-4-carboxamide ribonucleotide formyltransferase/IMP cyclohydrolase
11	PNP	4860	Purine nucleoside phosphorylase
12	BGLAP	632	Bone gamma-carboxyglutamate protein
13	TNFSF11	8600	TNF superfamily member 11
14	CAP1	10487	Cyclase associated actin cytoskeleton regulatory protein 1
15	KL	9365	Klotho
16	GPX1	2876	Glutathione peroxidase 1
17	GSN	2934	Gelsolin
18	TPI1	7167	Triosephosphate isomerase 1
19	TGF-β1	7040	Transforming growth factor beta 1
20	ANXA2	302	Annexin A2
21	TPM4	7171	Tropomyosin 4
22	VDR	7421	Vitamin D receptor
23	IDH2	3418	Isocitrate dehydrogenase (NADP( +))2
24	IGF1	3479	Insulin like growth factor 1
25	SOD2	6648	Superoxide dismutase 2
26	ESR1	2099	Estrogen receptor 1
27	SIRT1	23411	Sirtuin 1
28	ESR2	2100	Estrogen receptor 2
29	POMC	5443	Proopiomelanocortin
30	AR	367	Androgen receptor
31	IL6	3569	Interleukin 6
32	PKM	5315	Pyruvate kinase M1/2
33	COL1A2	1278	Collagen type I alpha 2 chain
34	GPD2	2820	Glycerol-3-phosphate dehydrogenase 2
35	LEP	3952	Leptin
36	REN	5972	Renin
37	P4HB	5034	Prolyl 4-hydroxylase subunit beta
38	TLN1	7094	Talin 1
39	GAPDH	2597	Glyceraldehyde-3-phosphate dehydrogenase
40	VCL	7414	Vinculin

**Table 2:** The list of 21 genes participated in the bone regeneration gathered from regeneration gene database.

No	Name	Entrez ID	Gene name
1	GSK3B	2932	Glycogen synthase kinase 3 beta
2	CSF3	1440	Colony stimulating factor 3
3	BDNF	627	Brain derived neurotrophic factor
4	MCAM	4162	Melanoma cell adhesion molecule
5	MMP2	4313	Matrix metallopeptidase 2
6	HGF	3082	Hepatocyte growth factor
7	PTGS2	5743	Prostaglandin-endoperoxide synthase 2
8	NGF	4803	Nerve growth factor
9	MMP9	4318	Matrix metallopeptidase 9
10	HIF1A	3091	Hypoxia inducible factor 1 subunit alpha
11	FGF2	2247	Fibroblast growth factor 2
12	BMP7	655	Bone morphogenetic protein 7
13	RUNX2	860	RUNX family transcription factor 2
14	BMP4	652	Bone morphogenetic protein 4
15	BMP2	650	Bone morphogenetic protein 2
16	SHH	6469	Sonic hedgehog signaling molecule
17	IL1B	3553	Interleukin 1 beta
18	FGF18	8817	Fibroblast growth factor 18
19	SPP1	6696	Secreted phosphoprotein 1
20	KDR	3791	Kinase insert domain receptor
21	TEK	7010	TEK receptor tyrosine kinase

**Figure 1:**
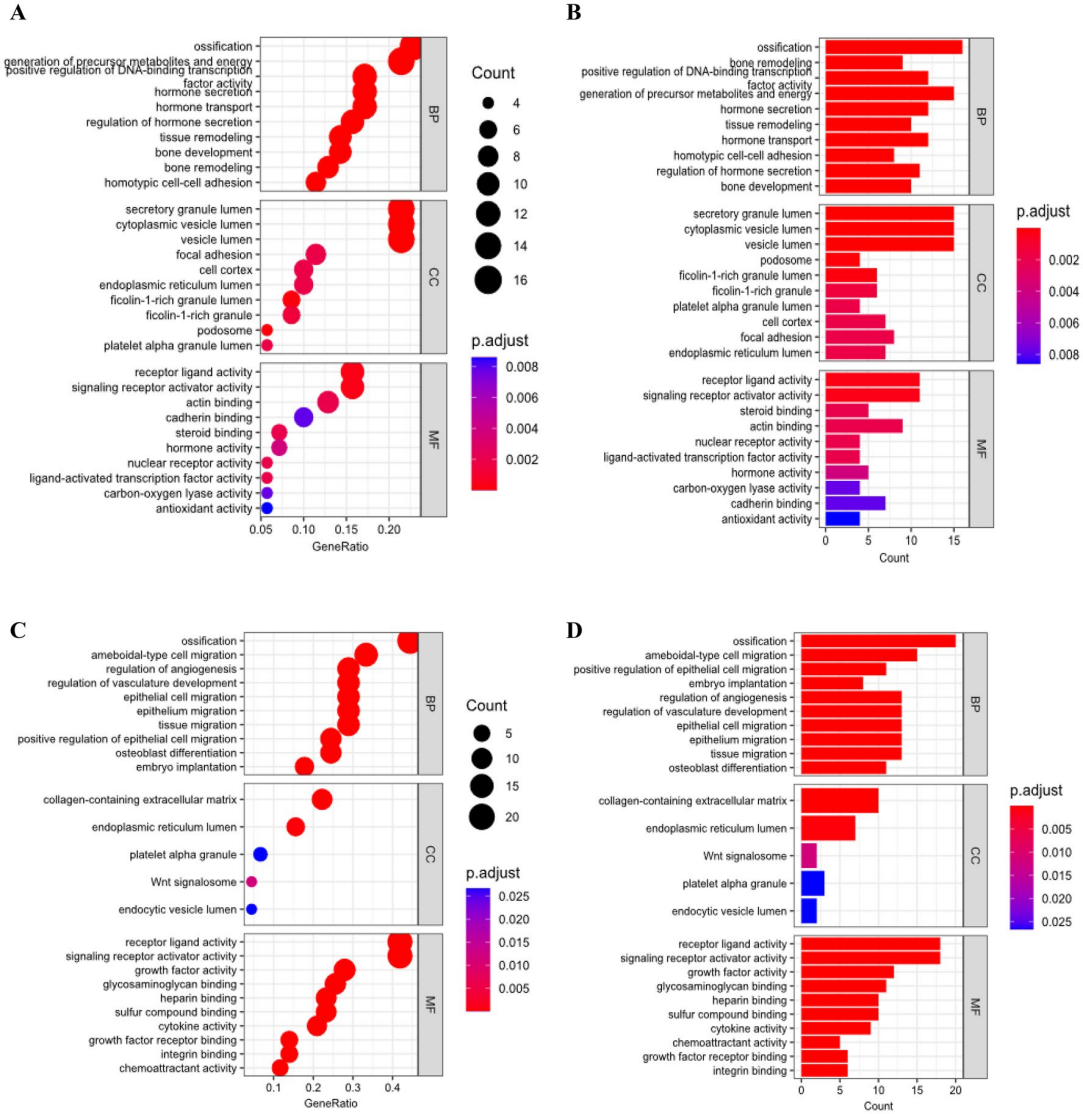
GO enrichment analysis of (**A**) OSP dot plot, (**B**) OSP bar plot, (**C**) BRP dot plot, and (**D**) BRP bar plot. OSP genes were primarily involved in biological processes such as ossification and the generation of precursor metabolites and energy, while BRP genes were mainly enriched in ossification and cell migration in biological processes.

GO enrichment analysis revealed distinct functional characteristics for the OSP and BRP genes (Fig. 1). OSP genes were primarily involved in biological processes such as ossification and the generation of precursor metabolites and energy. In terms of cellular localization, OSP genes were predominantly found in the secretory granule lumen, cytoplasmic vesicle lumen, and vesicle lumen. Moreover, OSP genes exhibited molecular functions related to receptor-ligand activity and signaling receptor activator activity.

**Figure 2:**
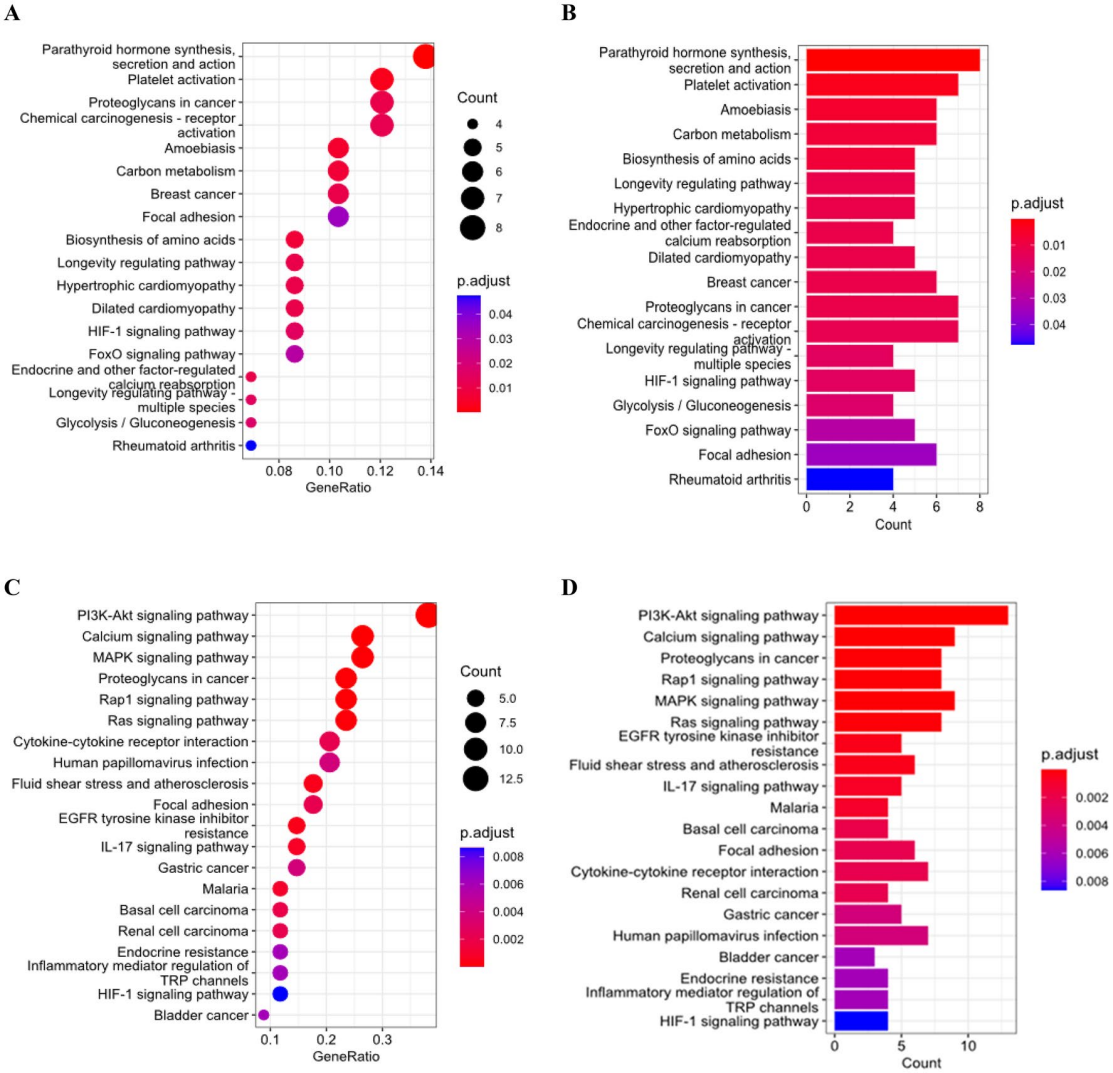
KEGG enrichment analysis of (**A**) OSP dot plot, (**B**) OSP bar plot, (**C**) BRP dot plot, and (**D**) BRP bar plot. OSP genes were significantly enriched in the parathyroid hormone synthesis and platelet activation pathways. In contrast, BRP genes were predominantly enriched in the PI3K–Akt signaling pathway, the calcium signaling pathway, and the MAPK signaling pathway^30,31^.

On the other hand, BRP genes were mainly enriched in ossification and cell migration in biological processes. In terms of cellular components, BRP genes were primarily associated with the collagen-containing extracellular matrix and endoplasmic reticulum lumen. Additionally, BRP genes displayed molecular functions related to receptor-ligand activity and signaling receptor activator activity. The KEGG pathway enrichment analysis further elucidated the specific pathways associated with the OSP and BRP genes (Fig. 2). OSP genes were significantly enriched in the parathyroid hormone synthesis and platelet activation pathways. In contrast, BRP genes were predominantly enriched in the PI3K-Akt signaling pathway, the calcium signaling pathway, and the MAPK signaling pathway.

These results suggest that the underlying mechanisms of OSP and BRP involve distinct biological processes, cellular components, molecular functions, and signaling pathways. OSP genes primarily contribute to bone formation and energy metabolism, while BRP genes are involved in processes such as cell migration and intracellular signaling.

### PPI and cluster

**Figure 3:**
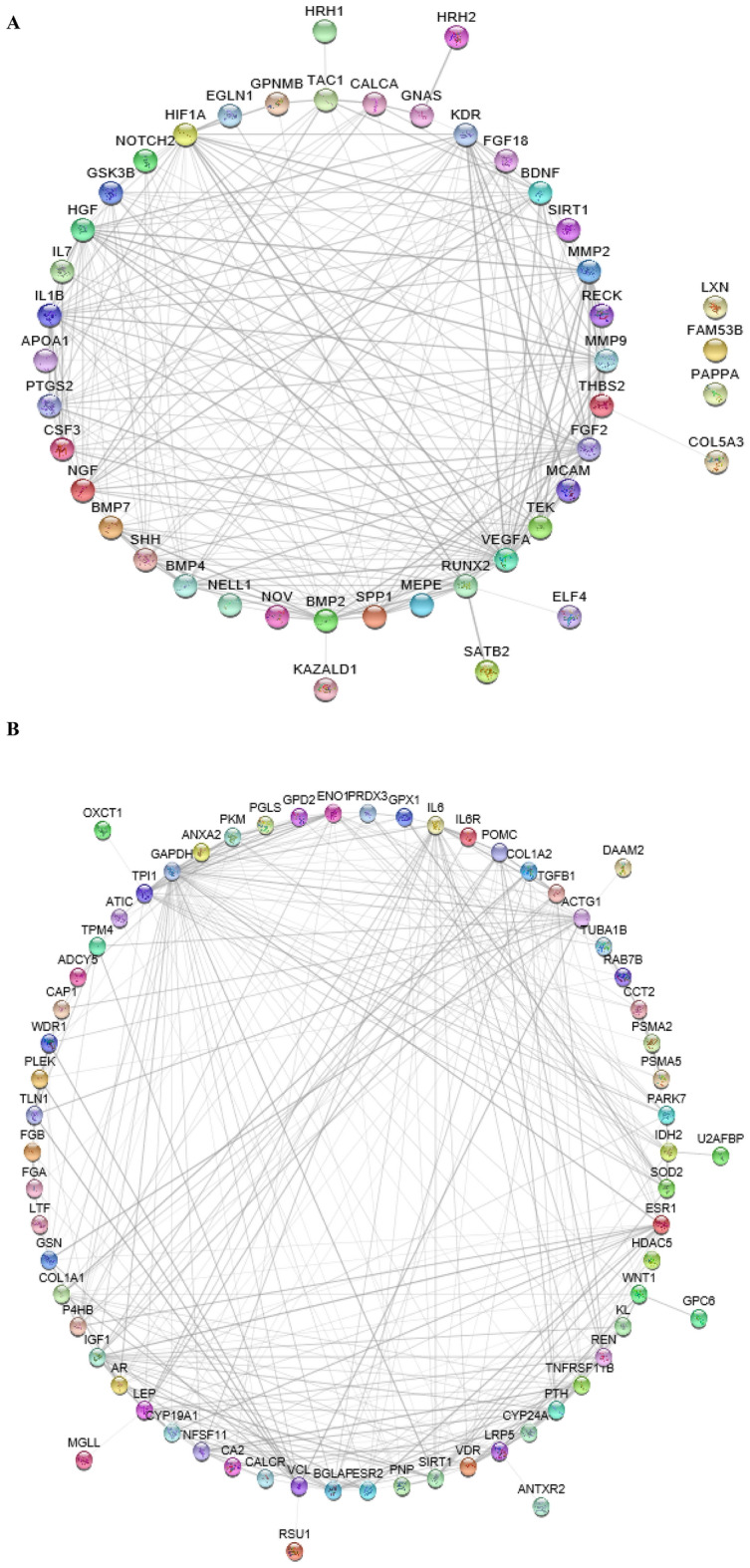
Protein–protein interaction (PPI) network analysis for OSP and BRP. (**A**) PPI network for BRP genes consists of 45 nodes (genes) and 241 edges (interactions). (**B**) PPI network for OSP genes consists of 70 nodes (genes) and 320 edges (interactions).

To investigate the PPI and identify functional clusters within the OSP and BRP gene sets, networks were constructed. The PPI network for BRP genes consisted of 45 nodes and 241 edges, indicating a substantial number of interactions among these genes. Similarly, the PPI network for OSP genes comprised 70 nodes and 320 edges, suggesting a complex network of interactions (Fig. 3).

**Figure 4:**
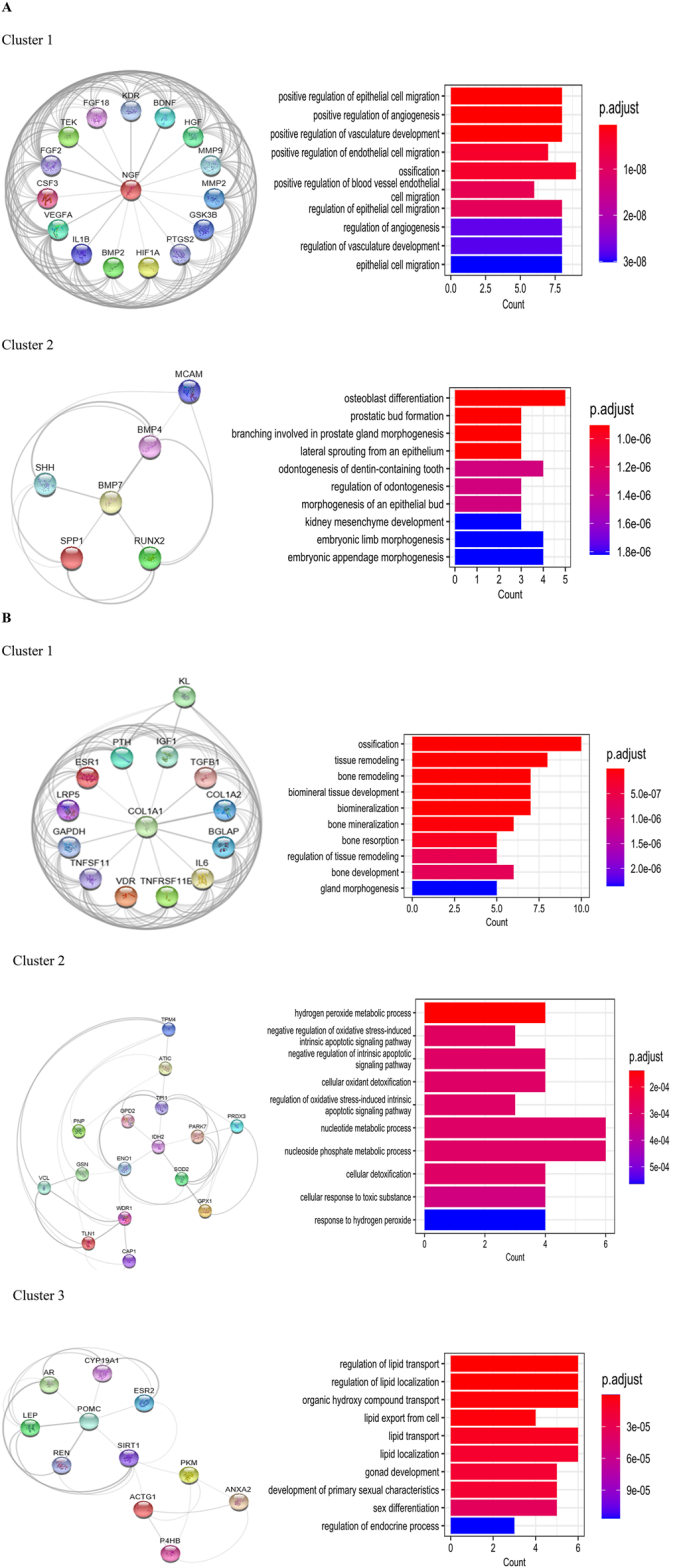
Significant PPI network clusters obtained by MCODE, and their corresponding enriched BP terms (p-value < 0.05) for (**A**) BRP and (**B**) OSP genes. In the BRP gene network, hub genes within clusters 1 and 2 were found to be significantly enriched in terms associated with ossification and osteoblast differentiation, indicating their potential roles in these processes. Similarly, in the OSP gene network, hub genes within clusters 1, 2, and 3 were significantly enriched in several biological processes. These included ossification, tissue remodeling, negative regulation of oxidative stress, and lipid transportation and localization.

To further analyze the PPI networks, cluster analysis was performed using MCODE. The cluster analysis revealed 2 distinct clusters within the BRP gene network and 3 clusters within the OSP gene network. These clusters represent groups of genes that exhibit higher connectivity and potential functional relationships. Enrichment analysis was conducted to assess the biological significance of the identified clusters. In the BRP gene network, hub genes within clusters 1 and 2 were found to be significantly enriched in terms associated with ossification and osteoblast differentiation, indicating their potential roles in these processes (Fig. 4).

Similarly, in the OSP gene network, hub genes within clusters 1, 2, and 3 were significantly enriched in several biological processes. These included ossification, tissue remodeling, negative regulation of oxidative stress, and lipid transportation and localization. These findings suggest the involvement of these hub genes in diverse biological processes related to OSP pathology. Overall, the PPI network and cluster analysis provided insights into the protein interactions and functional clustering of genes involved in BRP and OSP. The enrichment analysis highlighted specific biological processes associated with hub genes within the identified clusters.

### TFs-genes regulatory network

**Figure 5:**
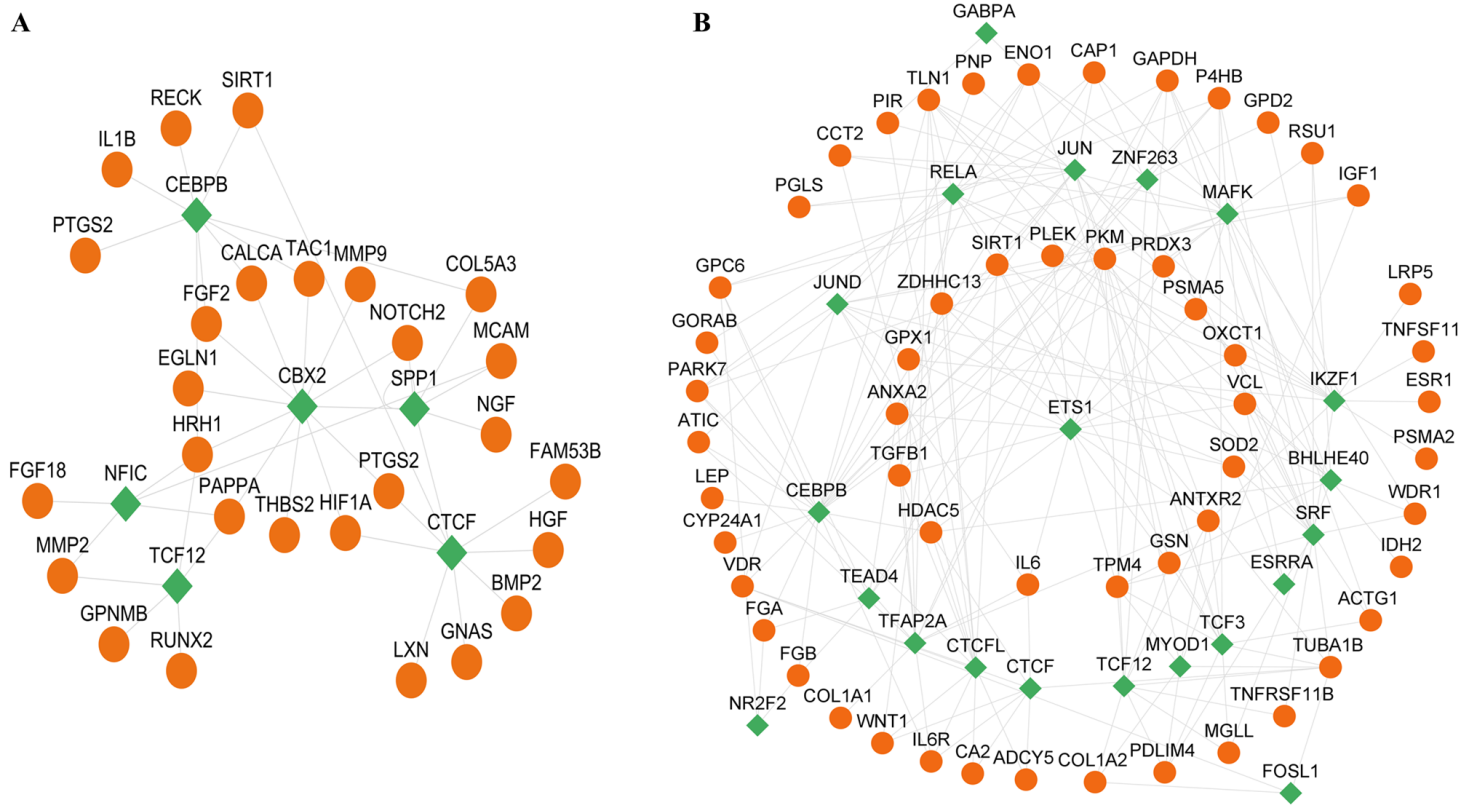
Interaction of TFs and target genes between (**A**) BRP genes and (**B**) OSP genes. The green color represents TFs and orange color represent genes. Among these TFs, CEBPB, CTCF, and TCF12 were found to be shared between the BRP and OSP genes, indicating their potential regulatory roles in both conditions.

To explore the regulatory network between TFs and genes involved in BRP and OSP, a TF-gene interaction analysis was performed. The top predicted TFs with a P-value < 0.05 were identified and are presented in Fig. 5. For BRP genes, the top TFs identified were CEBPB, CBX2, SPP1, CTCF, NFIC, and TCF12. On the other hand, the top TFs for OSP genes included GABPA, RELA, JUN, ZNF263, MAFK, JUND, CEBPB, TEAD4, TFAP2A, CTCFL, CTCF, TCF12, MYOD1, TCF3, ESRRA, SRF, BHLHE40, IKZF1, NR2F2, and FOSL1. Among these TFs, CEBPB, CTCF, and TCF12 were found to be shared between the BRP and OSP genes, indicating their potential regulatory roles in both conditions.

The CCAAT Enhancer Binding Protein Beta (CEBPB) is closely associated with bone cells and has been shown to impact bone mass regulation^50^. Dysregulated expression of CEBPB has been found to have differential effects on bone mass, suggesting its crucial role in bone metabolism and remodeling. Altered CEBPB activity may contribute to the development or progression of osteoporosis.

The CCCTC-binding factor (CTCF) is a zinc finger protein involved in DNA binding and the regulation of gene expression^51^. It plays a role in organizing chromatin structure, which influences the expression of target genes. In osteoblast primary cells, CTCF binding sites have been found near the promoter region of RUNX2, a master regulator of osteoblast differentiation^52^. This suggests that CTCF may participate in the regulation of osteoblast function and bone formation.

Also, transcription factor 12 (TCF12) is a transcription factor that regulates the differentiation of mesenchymal stromal cells (MSCs), which are progenitor cells involved in bone formation. Studies have demonstrated that TCF12 plays a role in osteogenesis and bone regeneration^53^. Its downregulation has been associated with enhanced bone regeneration, while its overexpression inhibits new bone formation by affecting BMP signaling. Thus, TCF12 may contribute to the balance between bone formation and resorption, which is crucial in maintaining bone health^54^.

The shared presence of CEBPB, CTCF, and TCF12 in the regulatory networks of both BRP and OSP genes suggests their potential involvement in the underlying mechanisms of both bone regeneration and osteoporosis. Understanding their regulatory roles and interactions with target genes can shed light on the molecular processes driving bone development, remodeling, and the pathological changes observed in osteoporosis.

### Choosing miRNAs and designing siRNAs

**Figure 6:**
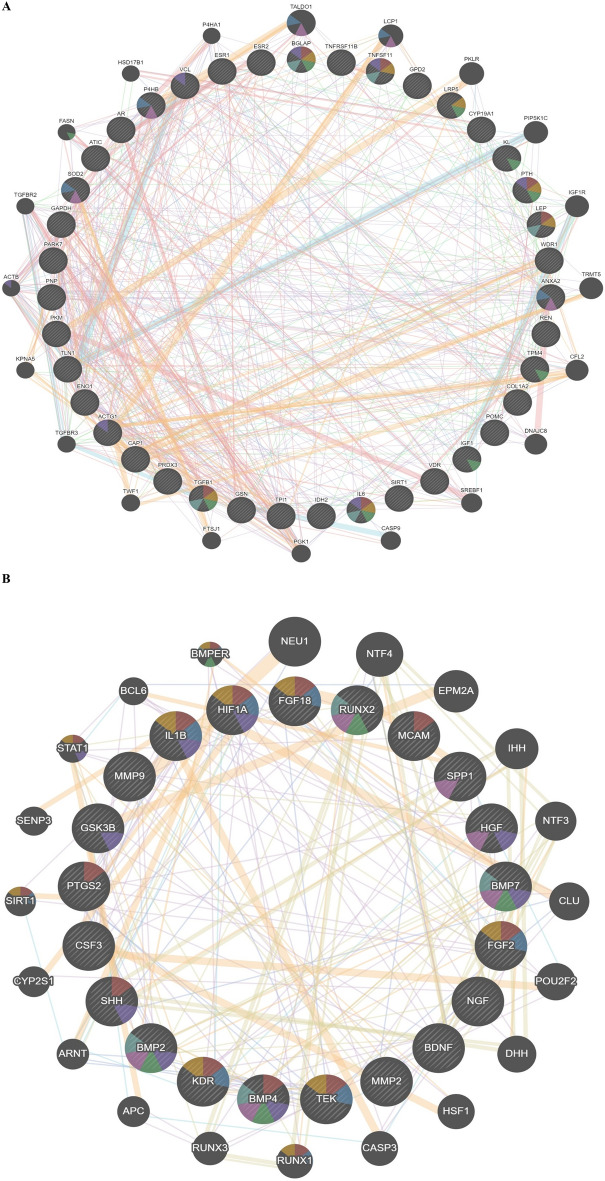
Gene ontology analyzing through GeneMANIA database. (**A**) OSP-involved genes, (**B**) BRP-involved genes. GeneMANIA database analysis identified five genes (BMP4, BMP2, BMP7, HIF1A, and IL1B) associated with ossification and angiogenesis and five genes (BGLAP, PTH, TGF-β1, TNFSF11, and IL6) involved in bone remodeling and ossification.

Based on the obtained results, GeneMANIA database analysis identified five genes (BMP4, BMP2, BMP7, HIF1A, and IL1B) associated with ossification and angiogenesis and five genes (BGLAP, PTH, TGF-β1, TNFSF11, and IL6) involved in bone remodeling and ossification (Fig. 6). GeneMANIA analysis revealed important genes associated with bone and osteoporosis. Among them, BMP4, BMP2, and BMP7 were identified as key regulators of bone development and remodeling, highlighting their role in promoting osteoblast differentiation and bone formation. Dysregulation of BMP signaling pathways has been linked to various bone disorders, including osteoporosis.

HIF1A, a hypoxia-inducible factor, was found to be associated with bone and angiogenesis. Its binding to the RANKL promoter enhances osteoclastogenesis, which can contribute to the bone loss observed in osteoporosis^55^. This highlights the importance of HIF1A in the bone remodeling process. Also, as IL1B, an inflammatory cytokine, is implicated in ossification, it promotes the production of RANKL, a key regulator of osteoclast differentiation and activity. Elevated IL1B levels have been associated with increased bone resorption and osteoporosis^56^.

Osteocalcin (BGLAP) serves as a marker of osteoblast activity and is critical for bone mineralization^57^. Parathyroid hormone (PTH) plays a crucial role in maintaining calcium homeostasis and bone remodeling^58^. Transforming growth factor beta 1 (TGF-β1) influences both osteoblast and osteoclast function, contributing to bone remodeling^59^. Moreover, TNFSF11 (RANKL) is a central mediator of osteoclastogenesis, and dysregulation of this gene can lead to bone loss^60^. Also, IL6, an inflammatory cytokine, has been linked to increased bone resorption and decreased bone formation in osteoporosis^61^.

The identified genes are interconnected in their roles and pathways related to bone and osteoporosis. BMPs, HIF1A, and IL1B can influence osteoclastogenesis and bone resorption through RANKL regulation^62^. BGLAP, PTH, TGF-β1, and TNFSF11 contribute to bone remodeling and the maintenance of bone mass. IL6, along with IL1B, promotes bone resorption processes. These findings emphasize the intricate relationships between these genes in bone biology and their relevance to osteoporosis. Further analysis using GeneMANIA and previous studies suggested that to promote bone regeneration, HIF1A, IL1B, TNFSF11, and IL6 should be downregulated, while BMP2, BMP4, BMP7, BGLAP, PTH, and TGF-β1 should be overexpressed.

To achieve gene regulation, miRNAs were predicted, and anti-miRNAs were designed to suppress the miRNAs' effect on target genes. MiRNA prediction was performed using the EnrichR database and Fischer's exact test, followed by validation using the Mirwalk database. Five anti-miRNAs were selected: has-miR-1277 targeting BMP4, has-miR-7155-5p targeting BMP2 and BMP7, has-miR-146a-5p targeting BGLAP and TGF-β1, has-miR-503 targeting PTH, and has-miR-542-3p targeting BMP7. Certain miRNAs, including miR-1277, miR-146a-5p, miR-503, and miR-542-3p, have been associated with bone metabolism and osteoporosis. miR-1277 is downregulated in osteoporotic bone tissue, potentially promoting osteoblast activity and bone formation. Conversely, miR-146a-5p is upregulated in osteoporosis, potentially enhancing osteoclast activity and bone resorption. Additionally, reduced expression of miR-503 and miR-542-3p in osteoporotic bone tissue suggests their involvement in osteoblast differentiation and bone formation. However, the specific role of miR-7155-5p in bone metabolism or osteoporosis remains unclear and requires further investigation.

**Figure 7:**
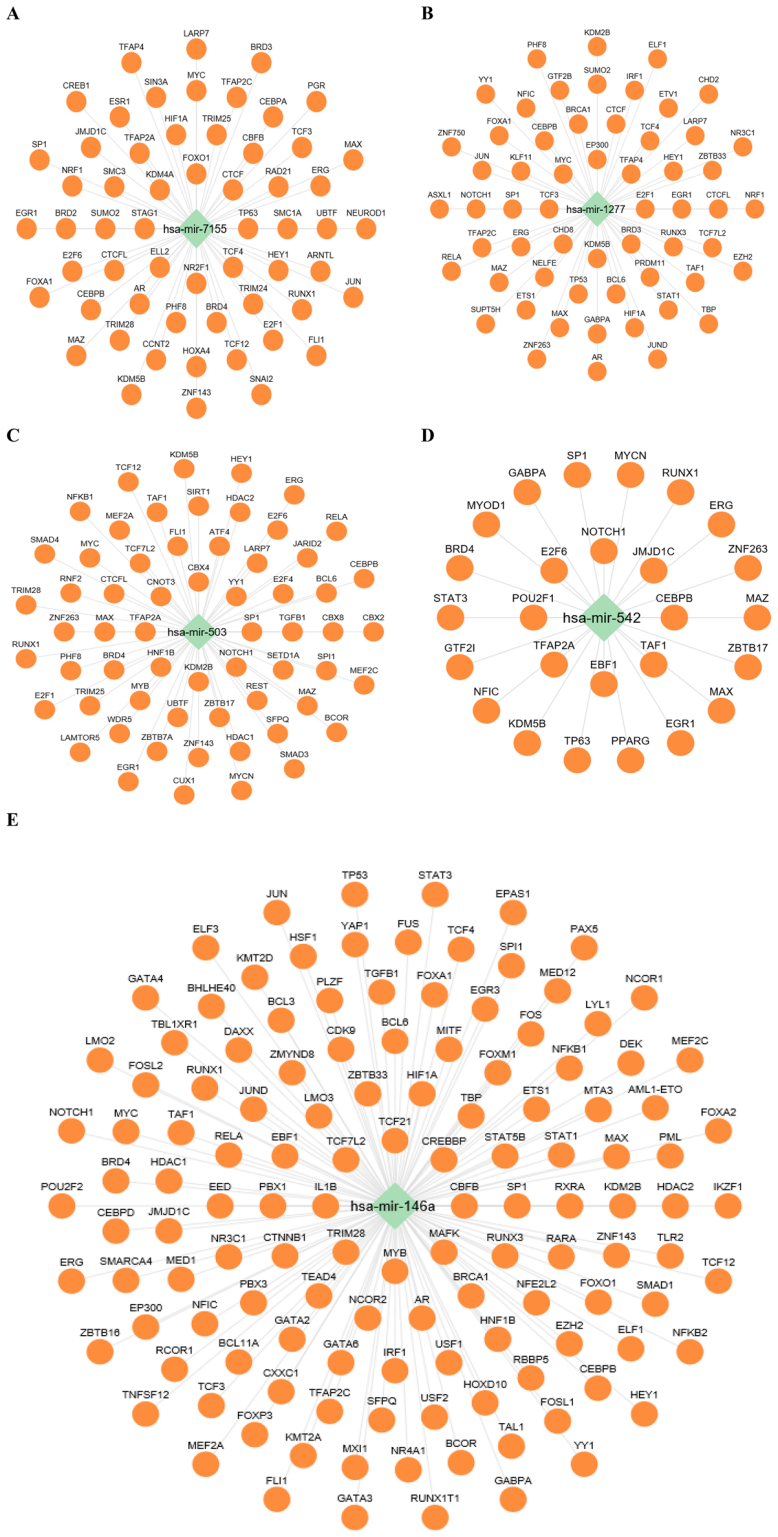
TFs involved in the regulation of (**A**) has-miR-7155, (**B**) has-miR-1277, (**C**) has-miR-503, (**D**) has-miR-542, and (**E**) has-miR-146a-5p. Green color represents miRNAs and orang color represents TFs. The regulatory network analysis identified TFs associated with these miRNAs. CTCF, CEBPA, TCF12, and HIF1A were found to regulate miR-7155-5p, while TNFSF11, IL1B, HIF1A, CEBPB, TCF12, and TGF-β1 were implicated in the regulation of miR-146a-5p.

In addition, TFs regulating these miRNAs (miR-1277, miR-7155-5p, miR-146a-5p, miR-503, and miR-542-3p) were explored using TransmiR^41^. The regulatory network analysis identified TFs associated with these miRNAs. CTCF, CEBPA, TCF12, and HIF1A were found to regulate miR-7155-5p, while TNFSF11, IL1B, HIF1A, CEBPB, TCF12, and TGF-β1 were implicated in the regulation of miR-146a-5p (Fig. 7). These TFs have well-established roles in bone metabolism and osteoporosis, further supporting the significance of these miRNAs in bone formation^13^.

To suppress the expression of the selected genes (HIF1A, IL1B, TNFSF11, and IL6), siRNAs were designed using Eurofins Genomic's siRNA design tool. The nucleotide sequences were retrieved from NCBI, and siRNAs with the best scores were chosen. The off-target effects of each siRNA were evaluated using the BLAST tool to select the most suitable siRNAs. Two anti-miRNAs (has-miR-7155-5p and has-miR-146a-5p) along with two designed siRNAs for HIF1A and TNFSF11 were selected as potential bone healing modulators (Table 3). These RNAi molecules can be further analyzed for their effectiveness when coupled with various carriers for delivery purposes. The selection of miRNAs and the design of siRNAs provide potential strategies for modulating gene expression to promote bone regeneration. Further experimental validation and delivery system optimization are necessary to assess their therapeutic potential in bone healing applications.

**Table 3:** anti-miRNA structures for has-miR-146a-5p and has-miR-7155-5p.

No	Target	miRNA	anti-miRNA	Length
1	BMP2/BMP7	hsa-miR-7155-5p	5'-GAUGGCCCAAGACCCCAGAGACAGAUGGCCCAAGACCCCAGA-3'	42
2	BGLAP/TGF-β1	hsa-miR-146a-5p	5'-AACCCAUGGAAUUCAGUUCUCAACAGAACCCAUGGAAUUCAGUUCUCA-3'	48

### MD simulations

**Figure 8:**
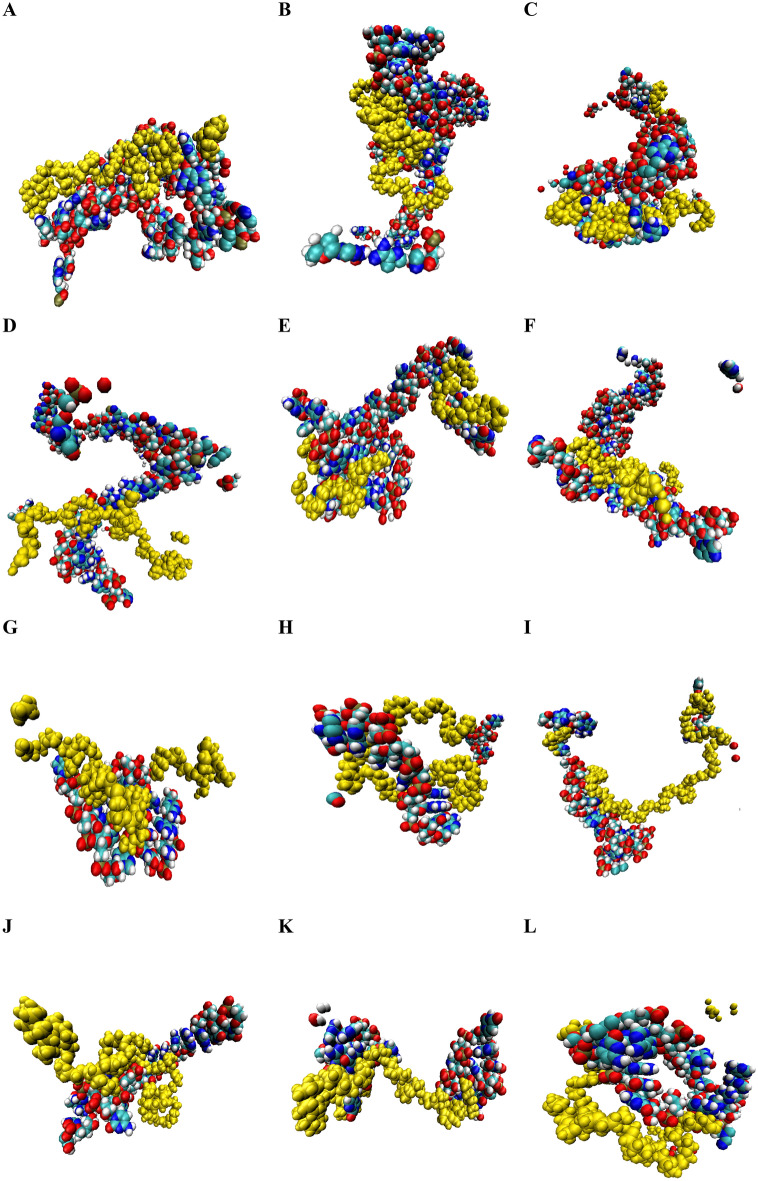
The snapshots of MD simulations among ant-miRNAs and siRNAs with PEG, PEI, and PEG-PEI. The output interaction of has-miR-146a-5p with (**A**) PEG, (**B**) PEI, and (**C**) PEG-PEI. The output interaction of has-miR-7155 with (**D**) PEG, (**E**) PEI, and (**F**) PEG-PEI. The output interaction of siRNA HIF1A with (**G**) PEG, (**H**) PEI, and (**I**) PEG-PEI. The output interaction of siRNA TNFSF11 with (**J**) PEG, (**K**) PEI, and (**L**) PEG-PEI. Yellow molecules represent the polymer.

**Figure 9:**
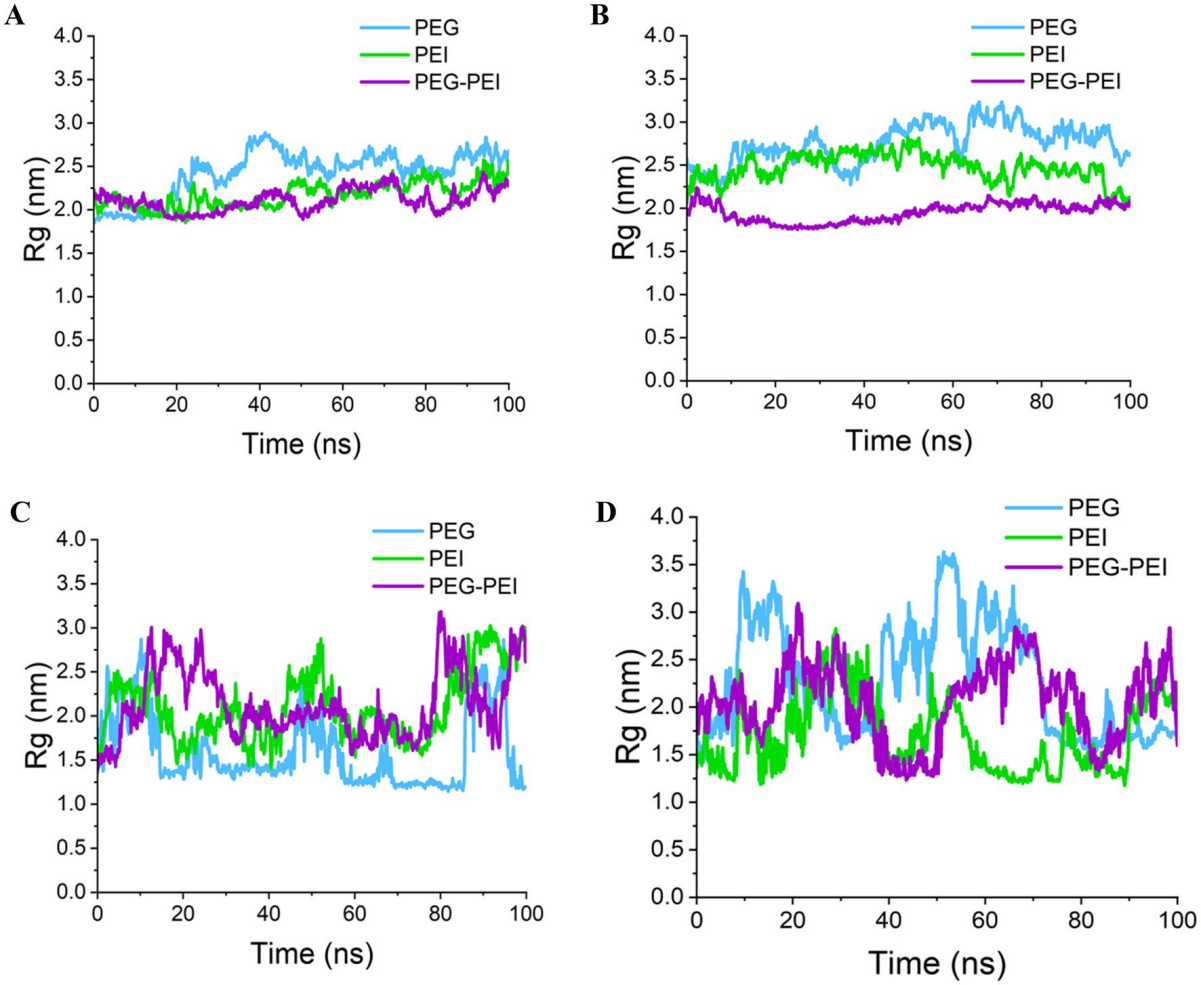
Radius of gyration of anti-miRNAs and siRNAs during MD simulation. (**A**) has-miR-146a-5p, (**B**) has-miR-7155, (**C**) HIF1A siRNA, (**D**) TNFSF11 siRNA. Employing PEG-PEI as a carrier for has-miR-146a-5p and has-miR-7155-5p, PEG for HIF1A, and PEI for TNFSF11 resulted in the formation of more compact polymer-RNA complexes.

In our study, we conducted comprehensive analyses to investigate the impact of different polymer types on RNA molecules under standardized conditions. Supplementary Fig. S2 represent the constructed nanocarriers in this study. Also, the outcomes were visually represented in Fig. 8, illustrating the interaction modes between various carrier types and RNAi molecules. To assess the behavior of each RNA, we calculated the ${R}_{g}$ over time, as depicted in Fig. 9. ${R}_{g}$ is a measure of the average distance between any point on the particle and its center of mass^63^. The equation used to compute the ${R}_{g}$ is as Eq. (1):

1 $${R}_{g}=\sqrt{\frac{1}{N}\sum_{i=1}^{N}|{r}_{i}-{r}_{com}{|}^{2}}$$

Here, N represents the total number of atoms in the system, ${r}_{i}$ denotes the position vector of atom i, and ${r}_{com}$ represents the position vector of the center of mass of the particle. We summarized the average ${R}_{g}$ values for each RNA in Table 4. Notably, employing PEG-PEI as a carrier for has-miR-146a-5p and has-miR-7155-5p, PEG for HIF1A, and PEI for TNFSF11 resulted in the formation of more compact polymer-RNA complexes.

**Table 4:** siRNAs designed to target specified genes.

No	Target	siRNA structure	Length
1	HIF1A	AUGGAACAUGAUGGUUCAC	19
2	TNFSF11	AAGGAAUUACAACAUAUCG	19

To investigate the conformational changes occurring during MD simulations, we employed the RMSD as a quantitative measure. The RMSD analysis offers valuable insights into the average spatial separation between two distinct groups of atoms within the system. By calculating the RMSD, we can gauge the extent of atomic fluctuations and ascertain whether the system has reached equilibrium conditions. This analysis yields a consistent value that reflects the overall displacement of atoms over time, thereby providing valuable information about the stability and dynamics of the molecular system^64^. The RMSD is computed using Eq. (2):

2 $$RMSD=\sqrt{\frac{1}{N}\sum_{i=1}^{N}{(|{r}_{i}\left(t\right)-{r}_{i}(0)|)}^{2}}$$

**Figure 10:**
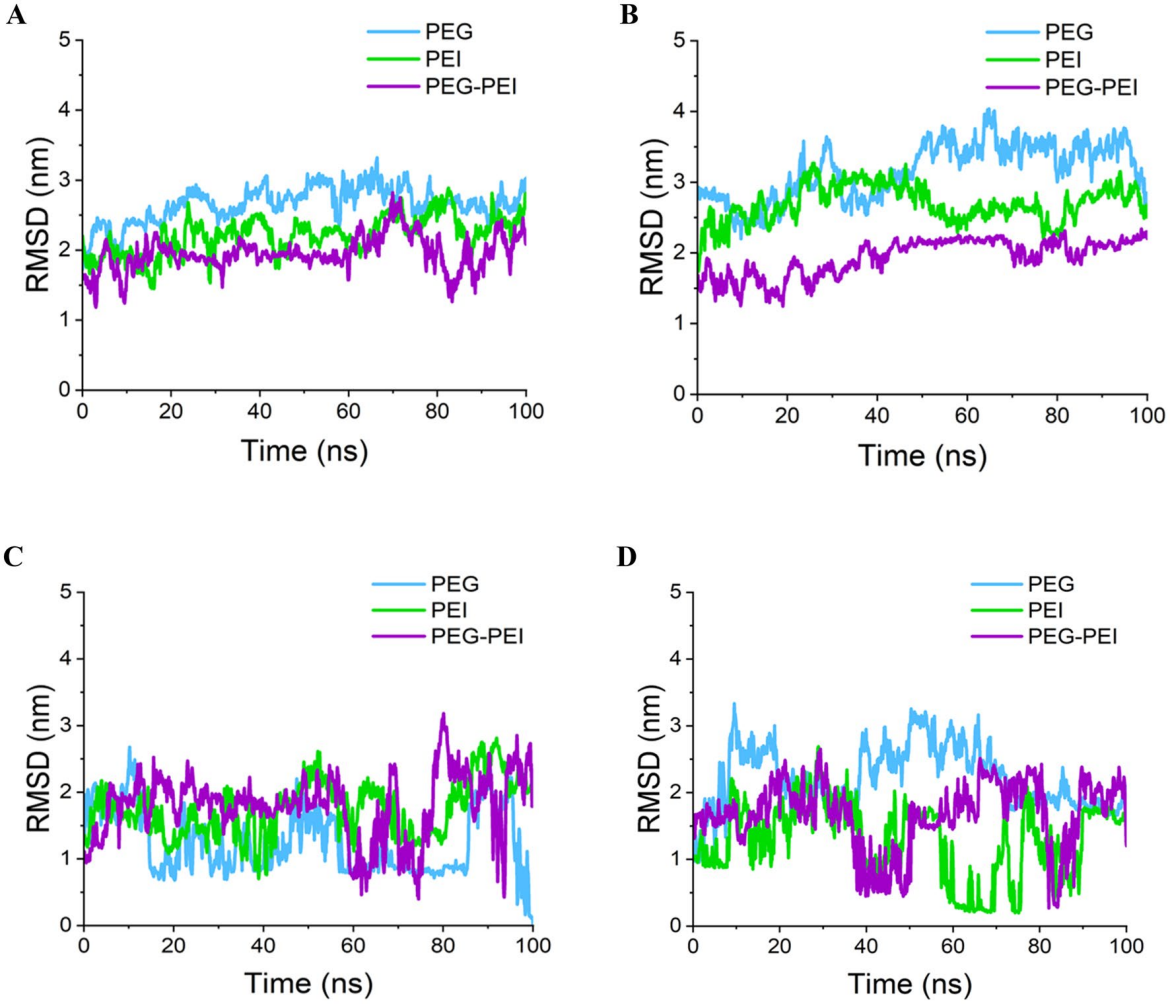
RMSD fluctuation of anti-miRNAs and siRNAs during MD simulation. (**A**) has-miR-146a-5p, (**B**) has-miR-7155, (**C**) HIF1A siRNA, (**D**) TNFSF11 siRNA. Results showed that PEG-PEI for has-miR-146a-5p and has-miR-7155-5p, as well as PEG for HIF1A and PEI for TNFSF11, exhibited lower fluctuations and higher stability.

Here, N represents the total number of atoms in the system, ${r}_{i}\left(t\right)$ denotes the current position vector of atom i at time t, and ${r}_{i}(0)$ represents its initial position vector. The RMSD analysis (Fig. 10) supported the trends observed in the ${R}_{g}$ analysis, indicating that PEG-PEI for has-miR-146a-5p and has-miR-7155-5p, as well as PEG for HIF1A and PEI for TNFSF11, exhibited lower fluctuations and higher stability. We have summarized these findings in Table 4.

**Figure 11:**
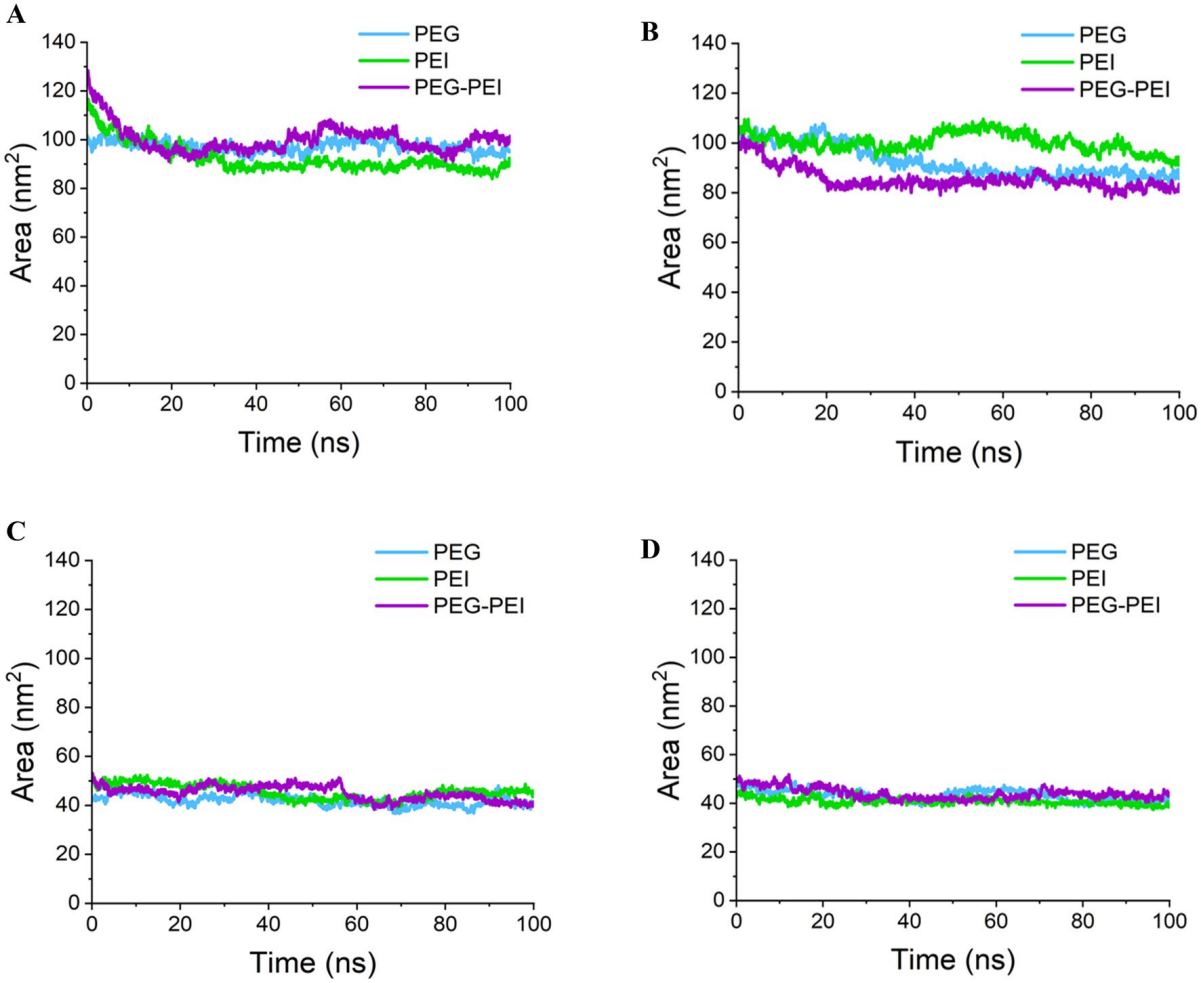
SASA of anti-miRNAs and siRNAs during MD simulation. (**A**) has-miR-146a-5p, (**B**) has-miR-7155, (**C**) HIF1A siRNA, (**D**) TNFSF11 siRNA. Integrating has-miR-146a-5p with PEI resulted in a lower SASA score compared to its interaction with PEG/PEI and PEG polymers.

The SASA of the RNAi molecules is influenced by factors such as RNA length, polymer ratio, and the hydrophilicity or hydrophobicity of the molecules. Higher SASA scores suggest greater exposure of RNAs to water, while lower scores indicate a higher degree of burial within the polymer^65^. Our results confirmed that PEG-PEI for has-miR-7155-5p, PEG for HIF1A, and PEI for TNFSF11 exhibited higher hydrophobicity. Moreover, integrating has-miR-146a-5p with PEI resulted in a lower SASA score compared to its interaction with PEG/PEI and PEG polymers (Fig. 11).

To deepen our understanding of RNAi molecules, we employed the Molecular Mechanics/Poisson-Boltzmann Surface Area (MMPBSA) approach within the GROMACS software to analyze their energy profiles. This approach allowed us to explore the contributions of vdW, electrostatic, and total resultant energies. It is important to note that the MMPBSA method, as implemented in GROMACS, primarily focuses on capturing the molecular mechanics and solvation energies associated with the binding process. However, it does not explicitly incorporate entropy contributions in the binding energy calculations^66^.

The vdW energy term provided valuable insights into the interplay between attractive and repulsive forces among non-bonded atoms. By assessing steric interactions and atomic overlap, we gained a deeper understanding of the spatial arrangements and potential clashes within the RNAi molecules. In parallel, the electrostatic energy term accounted for the interactions among charged atoms or groups. This allowed us to capture long-range electrostatic effects, discern regions of positive and negative charge distribution, and uncover electrostatic attractions or repulsions between different molecular components.

**Figure 12:**
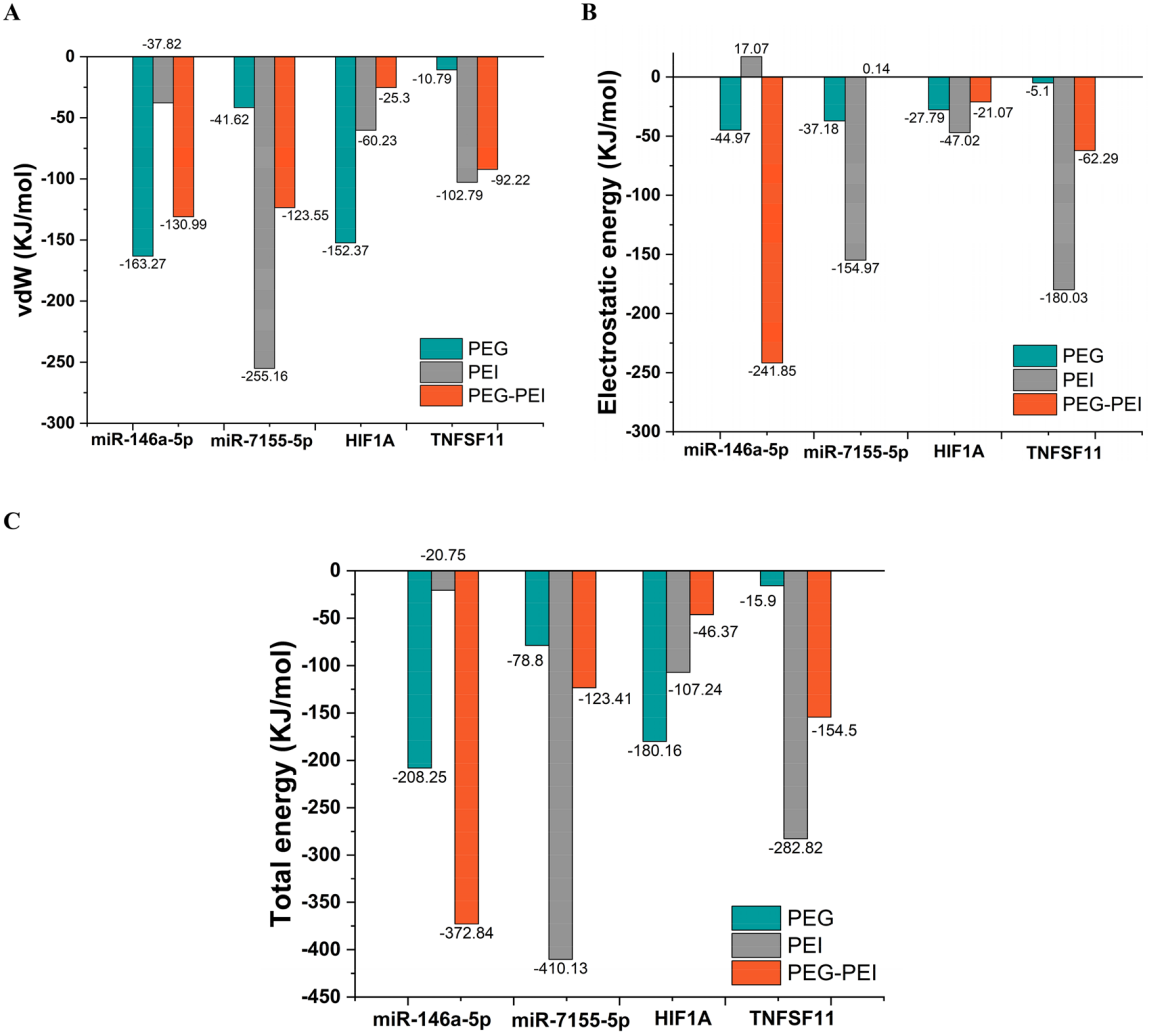
Energy analysis between RNAis and polymers. (**A**) Average van der Waals interaction energy of PEG, PEI, PEG-PEI with anti-miRNAs and siRNAs, (**B**) average electrostatic interaction energy of PEG, PEI, PEG-PEI with anti-miRNAs and siRNAs, and (**C**) average total interaction energy of PEG, PEI, PEG-PEI with anti-miRNAs and siRNAs.

By combining the vdW and electrostatic energy terms, we obtained the total resultant energy, serving as a comprehensive measure of the stability and strength of the RNAi molecules. Through our energy analysis, we further characterized the RNAi molecules, considering the vdW, electrostatic, and total resultant energies. These energy components proved pivotal in assessing stability. By calculating the average interaction energies between the polymers and RNAi over the simulation time, as shown in Fig. 12, we observed that PEG-PEI proved to be a suitable carrier for delivering has-miR-146a-5p, while PEI was effective for has-miR-7155-5p. Similarly, PEG was well-suited for delivering HIF1A, and PEI was advantageous for TNFSF11, as evidenced by lower energy levels and enhanced stability (Table 5). These findings collectively support the suitability of PEG-PEI, PEG, and PEI as carriers for delivering specific RNA molecules involved in bone formation and remodeling. The MD simulations provided valuable insights into the stability, conformational changes, hydrophobicity, and interaction energies of the polymer-RNA complexes, guiding the selection of optimal carriers for further investigation and potential therapeutic applications.

**Table 5:** The average of results among RNAis and different type of carriers.

Carrier	has-miR-146a-5p			has-miR-7155-5p			HIF1A			TNFSF11		
	PEG	PEI	PEG-PEI	PEG	PEI	PEG-PEI	PEG	PEI	PEG-PEI	PEG	PEI	PEG-PEI
vdW	−163.27	−37.82	−130.99	−41.62	−255.16	−123.55	−152.37	−60.23	−25.3	−10.79	−102.79	−92.22
Electrostatic energy	−44.97	17.07	−241.85	−37.18	−154.97	0.14	−27.79	−47.02	−21.07	−5.1	−180.03	−62.29
Total energy	−208.25	−20.75	−372.84	−78.8	−410.13	−123.41	−180.16	−107.24	−46.37	−15.9	−282.82	−154.5
SASA	97.1941	92.29	91.0163	92.41	82.3212	85.5460	41.9838	45.6116	45.0956	43.2122	40.7477	43.9826
Gyration radius	2.4297	2.1736	2.1084	2.7556	2.4739	2.9489	1.6069	2.1073	2.1339	2.2696	1.7092	2.0530
RMSD	2.6534	2.2128	1.9522	3.1690	2.6966	2.9361	1.2511	1.7515	1.7996	2.2100	1.2948	1.6635

## Discussion

This research focused on the development of antagomirs and siRNAs as potential therapeutic agents for modulating biological processes in osteoporosis and bone healing. Gene ontology analysis and systems biology techniques were employed to gather relevant information from various databases. Furthermore, the feasibility of utilizing polymeric bioresponsive nanocarriers for delivering the proposed siRNAs and miRNA antagonists was evaluated through molecular dynamics simulations. The findings from this study lay the foundation for further exploration and optimization of these novel therapeutic approaches in the field of bone-related disorders.

The integration of GO and KEGG pathway enrichment analyses revealed distinct functional characteristics and signaling pathways associated with BRP and OSP. The enrichment analysis identified key biological processes, cellular components, and molecular functions specific to each condition. OSP genes primarily contribute to bone formation, energy metabolism, and receptor-ligand signaling, while BRP genes are involved in processes such as cell migration and intracellular signaling. Furthermore, the PPI network analysis and cluster analysis provided a comprehensive view of the PPI and functional clustering within the BRP and OSP gene sets. The identified clusters represented groups of genes with higher connectivity and potential functional relationships. Enrichment analysis of these clusters revealed their significance in ossification, osteoblast differentiation, tissue remodeling, regulation of oxidative stress, and lipid transportation.

The shared TFs CEBPB, CTCF, and TCF12 in the OSP and BRP genes indicate their potential dual role in bone formation and remodeling, suggesting their involvement in osteoporosis. Dysregulation of these TFs can disrupt the delicate balance of bone remodeling, leading to impaired bone formation and increased susceptibility to osteoporosis. Targeting these TFs or their downstream signaling pathways holds promise as novel therapeutic strategies for preventing and treating osteoporosis. Our analysis sheds light on the complex transcriptional regulatory mechanisms underlying bone and osteoporosis, offering valuable targets for further investigation and potential therapeutic interventions.

Our analysis identified two microRNAs, miR-7155-5p and miR-146a-5p, with potential roles in bone formation. While miR-7155-5p's functions in bone cells require further investigation, miR-146a-5p has been extensively studied and found to be a suppressor of osteoblastogenesis and bone formation. Its expression increases with age and is associated with age-induced bone loss^67^. Deletion of miR-146a-5p has been shown to protect against bone loss in mice^68^. These findings suggest that targeting miR-146a-5p could be a promising strategy for mitigating osteoporosis.

The identification of TFs involved in regulating miRNAs associated with bone formation, particularly miR-7155-5p and miR-146a-5p, sheds light on the molecular mechanisms underlying osteoporosis. Dysregulation of bone remodeling processes is a key factor in osteoporosis, leading to decreased bone density and increased fracture risk. Among the TFs identified in the TF-miRNA regulatory network, HIF1A and TNFSF11 have known involvement in bone metabolism and regulation.

HIF1A, a critical factor in bone homeostasis, has been implicated in both the maintenance of bone balance and the development of osteoporosis. Through its interaction with specific regions in gene promoters, such as TNFSF11, HIF1A enhances the production of RANKL, a pivotal regulator of bone remodeling. This mechanism plays a significant role in postmenopausal osteoporosis, where excessive bone resorption occurs^55^. The dysregulation of TNFSF11/RANKL disturbs the delicate equilibrium between bone resorption and formation, contributing to the pathogenesis of osteoporosis.

In this study, we investigated the functional role of specific molecules, namely has-miR-7155-5p and has-miR-146a-5p anti-miRNAs, as well as siRNAs targeting HIF1A and TNFSF11, in the context of bone formation and remodeling. To effectively deliver these molecules, we assessed the performance of different carriers, namely PEG, PEI, and PEG-PEI. The goal was to identify the most suitable carrier for efficient delivery of these therapeutic agents in the context of bone-related processes.

The study found that PEG-PEI, PEG, and PEI exhibited stability for delivering has-miR-146a-5p, HIF1A, and TNFSF11, respectively, as indicated by low RMSD and ${R}_{g}$ fluctuations. The integration of has-miR-146a-5p with PEI resulted in a lower SASA score, indicating improved complex stability. Moreover, PEG-PEI, PEG, and PEI showed higher hydrophobicity for delivering has-miR-7155-5P, HIF1A, and TNFSF11, respectively. The analysis of energy levels also supported the enhanced stability of the carrier systems, with PEG-PEI, PEG, and PEI demonstrating lower energy levels. These findings highlight the potential of these carrier systems for efficient delivery and sustained release of therapeutic molecules in bone-related applications.

It is worth noting that previous studies have successfully developed a PEG-PEI Alginate hydrogel complex, which combined miR-146a/PEG-PEI nanoparticles with basic fibroblast growth factor (bFGF). This innovative approach utilized Alginate gel as a carrier and demonstrated the effectiveness of miR-146a/bFGF/PEG-PEI alginate hydrogel in promoting the proliferation and odontogenic differentiation of dental pulp cells (DPCs) in the presence of inflammation^69^. These findings support the notion that a well-designed PEG-PEI alginate hydrogel nanocomplex can create a favorable microenvironment for enhancing the tissue regeneration capability of DPCs in response to inflammation.

Furthermore, we discussed a previous study that introduced a novel cationic mixed micellar nanoparticle (MNP) system comprised of poly(ε-caprolactone)-block-poly(2-aminoethylethylene phosphate) (PCL-b-PPEEA) and poly(ε-caprolactone)-block-poly(ethylene glycol) (PCL-b-PEG) as a carrier for HIF-1α siRNA in the treatment of hypoxic tumors^70^. The MNP system demonstrated efficient delivery of siRNA, leading to the inhibition of HIF-1α expression and subsequent suppression of critical processes such as cell proliferation, migration, and angiogenesis under hypoxic conditions. These findings highlight the potential of the MNP system as a targeted therapeutic approach for addressing the challenges posed by the hypoxic tumor microenvironment.

In conclusion, our study underscores the importance of TFs and miRNAs in bone formation and the pathogenesis of osteoporosis. The interplay between TFs and miRNAs presents potential therapeutic targets for the treatment of osteoporosis and the improvement of bone health. These findings enhance our understanding of the molecular mechanisms involved in bone regeneration and osteoporosis, laying the groundwork for future research and the development of targeted interventions.

Additionally, our investigation highlights the efficacy of PEG-PEI, PEG, and PEI as carriers for delivering specific molecules implicated in bone formation and remodeling. These carrier systems exhibit promise for therapeutic applications in bone-related disorders. Nevertheless, further studies are necessary to validate their effectiveness in vivo and explore their broader impact on bone regeneration and remodeling processes. Overall, this study provides valuable insights into the intricate molecular landscape of bone biology and osteoporosis, offering potential avenues for therapeutic interventions and advancing our comprehension of bone healing and disease management.

### Supplementary Information

Supplementary Figures.

## Data Availability

All the data that was used in this study are presented along with the findings. The 21 bone regeneration-involved genes were identified utilizing the regeneration gene database (http://regene.bioinfo-minzhao.org/), while the genes associated with osteoporosis disease were identified using the Disgenet database (https://www.disgenet.org/) with a score > 0.3, which includes 40 genes.

## Abbreviations

bFGF: Basic fibroblast growth factor
BMD: Bone mineral density
BMPs: Bone morphogenetic proteins
BMP4: Bone morphogenetic protein 4
BRP: Bone regeneration
CEBPB: CCAAT Enhancer Binding Protein Beta
CTCF: CCCTC-binding factor
Cbfa1: Core-binding factor alpha-1
DPCs: Differentiation of dental pulp cells
GO: Gene ontology
MSCs: Mesenchymal stromal cells
miRNA: MicroRNA
MNP: Mixed micellar nanoparticle
MCODE: Molecular Complex Detection
MMPBSA: Molecular Mechanics/Poisson–Boltzmann Surface Area
MD: Molecular dynamics
OSP: Osteoporosis
PTH: Parathyroid hormone
PME: Particle Mesh Ewald
PEG: Polyethylene glycol
PEI: Polyethyleneimine
PPI: Protein–protein interaction
Rg: Radius of gyration
Runx2: Runt-related transcription factor 2
RNAi: Ribonucleic acid interference
RMSD: Root-mean-square deviation
siRNA: Small interfering RNA
SASA: Solvent-accessible surface area
TCF12: Transcription factor 12
TFs: Transcription factors
TGF-β1: Transforming growth factor beta 1
vdW: Van der Waals

## Code availability

The protein–protein interaction (PPI) network of genes was constructed using the STRING database (https://string-db.org/), while the PPI network was visualized using Cytoscape v.3.9.0 software (https://cytoscape.org/). The GO term and KEGG pathway enrichment analyses were performed based on the C*luster Profiler* function package of the R language (https://github.com/YuLab-SMU/clusterProfiler) to identify the key pathways (p-value < 0.05 was considered significant). Then, the results were visualized using the ggplot2 R package (https://ggplot2.tidyverse.org/).
