# Author Correction: In silico engineering and simulation of RNA interferences nanoplatforms for osteoporosis treating and bone healing promoting

**DOI:** 10.1038/s41598-024-53032-0

**Published:** 2024-02-05

**Authors:** Aylar Imanpour, Hanieh Kolahi Azar, Dorna Makarem, Zeinab Nematollahi, Reza Nahavandi, Mohammadreza Rostami, Nima Beheshtizadeh

**Affiliations:** 1 Regenerative Medicine Group (REMED), Universal Scientific Education and Research Network (USERN), Tehran, Iran; 2 Department of Pathology, Tabriz University of Medical Sciences, Tabriz, Iran; 3 Escuela Tecnica Superior de Ingenieros de Telecomunicacion, Politecnica de Madrid, Madrid, Spain; 4 UCL Department of Nanotechnology, Division of Surgery and Interventional Science, University College London, London, UK; 5 Department of Biochemical and Pharmaceutical Engineering, School of Chemical Engineering, College of Engineering, University of Tehran, Tehran, 11155-4563 Iran; 6 Food Science and Nutrition Group (FSAN), Universal Scientific Education and Research Network (USERN), Tehran, Iran; 7 Division of Food Safety and Hygiene, Department of Environmental Health Engineering, School of Public Health, Tehran University of Medical Sciences, Tehran, Iran; 8 Department of Tissue Engineering, School of Advanced Technologies in Medicine, Tehran University of Medical Sciences, Tehran, Iran

Correction to: *Scientific Reports* 10.1038/s41598-023-45183-3, published online 24 October 2023

The original version of this Article contained an error in the spelling of the author Nima Beheshtizadeh, which was incorrectly given as Nima Behestizadeh.

The original Article has been corrected.

