# Supplementary material for: In silico engineering and simulation of RNA interferences nanoplatforms for osteoporosis treating and bone healing promoting
Source: Sci Rep. 2023 Oct 24;13:18185. doi: 10.1038/s41598-023-45183-3 (PMC10598124; doi:10.1038/s41598-023-45183-3)
Supplement: Supplementary file 1 — Supplementary Figures. [file 41598_2023_45183_MOESM1_ESM.docx]

In silico engineering and simulation of RNA interferences nanoplatforms for osteoporosis treating and bone healing promoting

Aylar Imanpour^1^, Hanieh Kolahi Azar^1, 2^, Dorna Makarem^3^, Zeinab Nematollahi^4^, Reza Nahavandi^5^, Mohammadreza Rostami ^1, 6^, Nima Behestizadeh^1, 7*^

*1. Regenerative Medicine group (REMED), Universal Scientific Education and Research Network (USERN), Tehran, Iran*

*2. Department of Pathology, Tabriz University of Medical Sciences, Tabriz, Iran*

*3. Escuela tecnica superior de ingenieros de telecomunicacion, Politecnica de Madrid, Madrid,España*

*4. UCL Department of Nanotechnology, Disvision of Surgery and interventional Science, University College London, London, UK*

*5. Department of Biochemical and Pharmaceutical Engineering, School of Chemical Engineering, College of Engineering, University of Tehran, Tehran, 11155-4563, Iran*

*6. Food Science and Nutrition Group (FSAN), Universal Scientific Education and Research Network (USERN), Tehran, Iran*

*7. Department of Tissue Engineering, School of Advanced Technologies in Medicine, Tehran University of Medical Sciences, Tehran, Iran*

* Corresponding author:

Dr. Nima Beheshtizadeh

Address: No. 88, Italia St, Qods Ave, Keshavarz Blvd, Tehran, 14177-55469, Iran.

Email: [n-beheshtizadeh@razi.tums.ac.ir](mailto:n-beheshtizadeh@razi.tums.ac.ir)


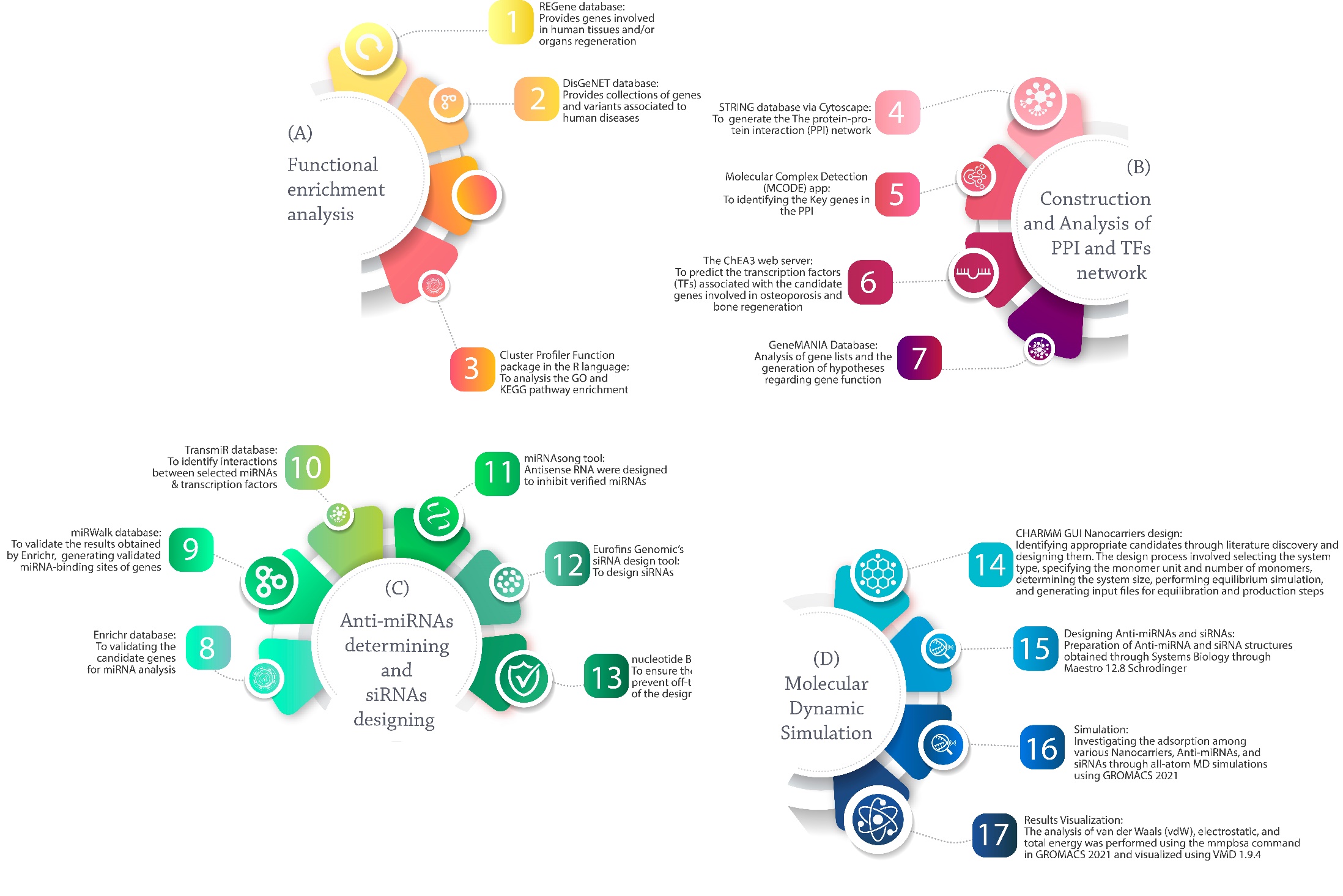


**Figure S1**. The flow chart of study procedures consists of 17 various steps: (A) Functional enrichment analyses, (B) Construction and analysis of PPI and TFs network, (C) Anti-miRNAs determining and siRNAs designing, and (D) Molecular dynamic simulation. Each step includes various procedures indicated in the chart.

| PEG | 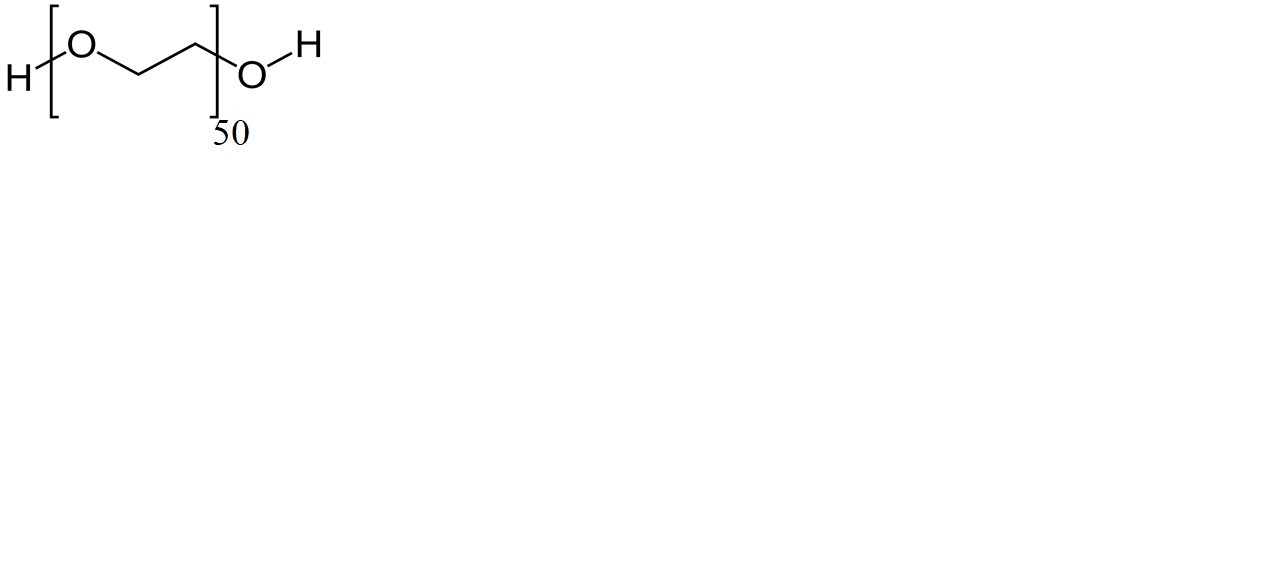 | 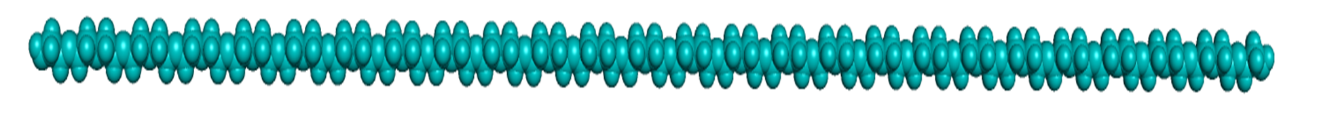 |
| --- | --- | --- |
| PEI | 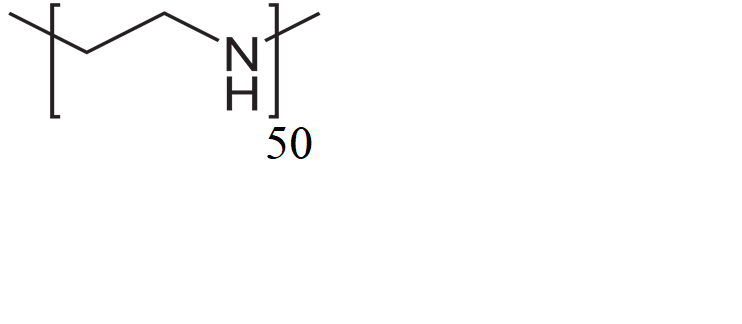 | 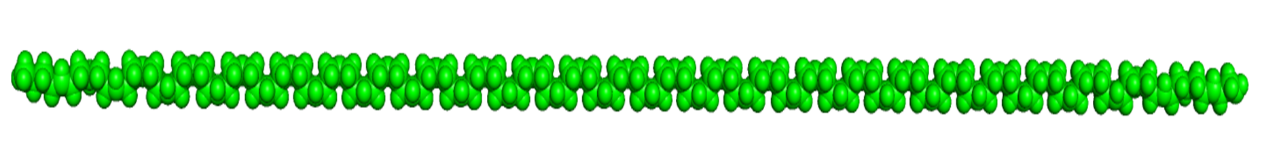 |
| PEG-PEI | 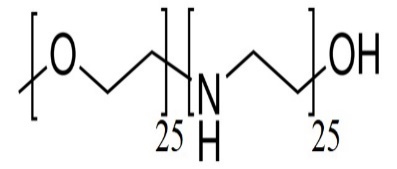 | 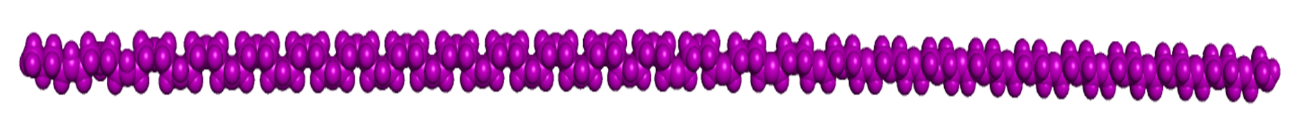 |

**Figure S2**. CHARM-GUI generated structure of these nanocarriers
